# Modulation of Hippocampal Gamma Oscillations by Dopamine in Heterozygous Reeler Mice *in vitro*

**DOI:** 10.3389/fncel.2019.00586

**Published:** 2020-02-11

**Authors:** Lu Wang, Dandan Zhao, Mengmeng Wang, Yuan Wang, Martin Vreugdenhil, Juntang Lin, Chengbiao Lu

**Affiliations:** ^1^The International-Joint Lab for Non-Invasive Neural Modulation, Xinxiang Medical University, Xinxiang, China; ^2^Key Laboratory for the Brain Research of Henan Province, Xinxiang Medical University, Xinxiang, China; ^3^Department of Neurobiology and Physiology, Xinxiang Medical University, Xinxiang, China; ^4^Department of Life Science, School of Health Sciences, Birmingham City University, Birmingham, United Kingdom; ^5^School of Biomedical Engineering, Xinxiang Medical University, Xinxiang, China

**Keywords:** dopamine, γ oscillation, hippocampus, NMDAR, PI3 kinase, reelin

## Abstract

The reelin haploinsufficient heterozygous reeler mice (HRM), an animal model of schizophrenia, have altered mesolimbic dopaminergic pathways and share similar neurochemical and behavioral properties with patients with schizophrenia. Dysfunctional neural circuitry with impaired gamma (γ) oscillation (30–80 Hz) has been implicated in abnormal cognition in patients with schizophrenia. However, the function of neural circuitry in terms of γ oscillation and its modulation by dopamine (DA) has not been reported in HRM. In this study, first, we recorded γ oscillations in CA3 from wild-type mice (WTM) and HRM hippocampal slices, and we studied the effects of DA on γ oscillations. We found that there was no difference in γ power between WTM and HRM and that DA increased γ power of WTM but not HRM, suggesting that DA modulations of network oscillations in HRM are impaired. Second, we found that *N*-methyl-D-aspartate receptor (NMDAR) antagonist MK-801 itself increased γ power and occluded DA-mediated enhancement of γ power in WTM but partially restored DA modulation of γ oscillations in HRM. Third, inhibition of phosphatidylinositol 3-kinase (PI3K), a downstream molecule of NMDAR, increased γ power and blocked the effects of DA on γ oscillation in WTM and had no significant effect on γ power but largely restored DA modulation of γ oscillations in HRM. Our results reveal that impaired DA function in HRM is associated with dysregulated NMDAR–PI3K signaling, a mechanism that may be relevant in the pathology of schizophrenia.

## Introduction

Reelin, a glycoprotein of the extracellular matrix, controls cell migration and layering in the developing brain, promotes the formation of synaptic circuits, and regulates synaptic transmission and plasticity in the postnatal and adult brain (Campo et al., 2009; Hwa and Gabriella, 2016). During development, reelin is expressed by the Cajal–Retzius cells in the hippocampus and cortex and granule cells in the cerebellum, whereas in the adult brain, reelin is secreted by GABAergic interneurons in the cortex and hippocampus (Ogawa et al., 1995; Knuesel, 2010; Brosda et al., 2011; Yuki et al., 2015).

The reelin gene is a susceptibility factor for early-onset psychiatric disorders, such as schizophrenia and autism. The heterozygous reeler mice (HRM) have a single reelin allele deficiency (Tueting et al., 2008). Similar to the brain of patients with schizophrenia, that of HRM exhibits a marked reduction in reelin, glutamic acid decarboxylase 67, dendritic spines, and synaptic function in the cortex and hippocampus (Tueting et al., 1999, 2006; Costa et al., 2001; Liu et al., 2001; Nullmeier et al., 2011), and abnormal behaviors, including impaired visual attention (Brigman et al., 2006), increased motor impulse (Ognibene et al., 2007), and persistent behavior (Macrì et al., 2010). Interestingly, reelin supplementation restored sensory motor gating and synaptic plasticity and reduced association learning deficits in HRM (Rogers et al., 2013) and schizophrenia-like symptoms (Ishii et al., 2015).

The hippocampal CA3 region plays a specific role in memory processes (Cherubini and Miles, 2015) and attention (Vinogradova, 2001; Bygrave et al., 2019) and controls dopamine (DA) release by forming a functional circuit in the ventral tegmental area (Luo et al., 2011). Extensive recurrent axon collaterals of CA3 pyramidal neurons connected with neighboring neurons, including GABAergic interneurons, compose a local circuit, and the interaction of pyramidal neurons and interneurons within the circuit generates synchronized activity, such as gamma (γ) frequency oscillations (30–80 Hz) (Traub and Wong, 1982; Bartos et al., 2007). γ oscillations are able to synchronize local, inter-region, or long-range neuronal activity and to promote information exchanges between neurons (Colgin, 2011; Fries, 2015) and are associated with higher brain function, such as attention, perceptual binding, learning, and memory (Womelsdorf and Fries, 2007; Buzsaki and Wang, 2012).

Schizophrenia has been suggested to be caused by the failure of integrating local and distributed neural circuits (Andreasen, 2000; Lee et al., 2003; Gallinat et al., 2004; Spencer, 2011; Jadi et al., 2016). In fact, studies have found that abnormal γ oscillations are associated with multiple symptoms of schizophrenia, such as hallucinations and delusion (Lee et al., 2003; Spencer et al., 2004). Schizophrenia is known to be associated with altered DA level (Winterer and Weinberger, 2004; Toda and Abi-Dargham, 2007), which influences information processes underlying cognitive process and may contribute to abnormal γ oscillations observed in patients with schizophrenia.

As an animal model of schizophrenia, HRM shows abnormal dopaminergic function, including reduced DA D1 and D2 receptors (D1R and D2R) in the striatum, reduced D1R- and D2R-mediated locomotor response (Matsuzaki et al., 2007), and increased expression of D2R in the striatum (Varela et al., 2015); and it also shows altered dopaminergic fiber densities in different brain areas, such as increase in the densities of tyrosine hydroxylase-immunoreactive (TH-IR) neurons in the hippocampus but decrease TH-IR neurons in the shell of the nucleus accumbens (Nullmeier et al., 2014). DA modulates fast network oscillations in the γ frequency band of rat hippocampus (Andersson et al., 2012) and beta frequency band of the mouse anterior cingulate cortex (Steullet et al., 2014); however, little is known about DA modulation of network oscillations in HRM.

Dopamine modulation of γ oscillations in rat hippocampus is involved in *N*-methyl-D-aspartate receptor (NMDAR)-dependent mechanism (Andersson et al., 2012). Methamphetamine, a psychostimulant, known to induce a strong DA release, enhances γ oscillations recorded in rat hippocampal slices also involved in NMDAR activation (Li et al., 2019). Studies have demonstrated that NMDAR is dysfunctional in schizophrenia. The cortical hyperexcitability and reduced function of NMDAR in parvalbumin-expressing inhibitory interneurons in schizophrenia are associated with increased γ activity (Spencer, 2011). A single dose of an application of the NMDAR antagonist MK-801 induces psychotic symptoms in humans and schizophrenia-like phenotype in animals, increases peak power, and reduces peak frequency of γ oscillations (Carlen et al., 2012; Lemercier et al., 2017).

Reelin increases NMDAR-dependent synaptic transmission and plasticity in the postnatal hippocampus (Qiu et al., 2006). Reelin deficiency causes increased expression of NR2A and NR2B of NMDAR subunits in the hippocampus from HRM (Isosaka et al., 2006). Blocking reelin secretion rapidly changes the subunit composition of NMDAR to a predominance of NR2B-containing NMDAR in cultured hippocampal neurons (Campo et al., 2009). The altered expression of NMDAR subunits may contribute to the modulation of network oscillations of HRM.

By binding to apolipoprotein E receptor 2 and very-low-density lipoprotein receptor (ApoER2/VLDLR), reelin activates different signaling cascades, one of which is phosphatidylinositol 3-kinase (PI3K) signaling pathway, and increases synaptic transmission by enhancing PI3K-dependent postsynaptic AMPAR insertion (Qiu et al., 2006; Ishii et al., 2016). PI3K is one of the downstream molecules in NMDAR activation, in which calcium influx through the NR2B subunit of NMDAR leads to the activation of PI3K (Brennan-Minnella et al., 2013). A previous study shows that nicotinic modulation of hippocampal γ oscillations involves PI3K activation (Wang et al., 2017). These studies indicate that PI3K may be involved in the modulation of γ oscillations in HRM.

In this study, we investigated γ oscillation and its modulation by DA in HRM using extracellular field potential recording to determine whether there are altered dopaminergic modulations of γ oscillation in HRM and the possible mechanisms associated with it.

## Materials and Methods

### Experimental Animals

Wild-type (WT) mice (c57BL/6N) and HRM (reelin+/−), 3- to 6-month-old male and female mice, were purchased from Model Animal Research Center of Nanjing University. WT animals used in this study are littermates of HRM. Mice were kept in standard housing conditions, with normal chow and water *ad libitum*, under a normal 6 AM light–6 PM dark cycle. The animals were anesthetized by intraperitoneal injection of Sagatal (pentobarbital sodium, 100 mg kg^–1^, Rhône Mérieux Ltd., Harlow, United Kingdom). When all pedal reflexes were abolished, the animals were perfused intracardially with chilled (4°C), oxygenated artificial cerebrospinal fluid (ACSF), in which sodium chloride had been replaced by iso-osmotic sucrose. This sucrose-ACSF contained (in mM) the following: 225 sucrose, 3 KCl, 1.25 NaH_2_PO_4_, 24 NaHCO_3_, 6 MgSO_4_, 0.5 CaCl_2_, and 10 glucose (pH 7.4). Horizontal slices (350 μm) of mouse brain containing the ventral hippocampus were cut at 4°C in sucrose-ACSF, using a Leica VT1000S vibratome (Leica Microsystems UK, Milton Keynes, United Kingdom), and stored at room temperature at the interface between recording of ACSF and humidified carbogen (95% O_2_–5% CO_2_) until these transferred to the recording chamber. The recorded ACSF contained (in mM) the following: 126 NaCl, 3 KCl, 1.25 NaH_2_PO_4_, 24 NaHCO_3_, 2 MgSO_4_, 2 CaCl_2_, and 10 glucose (pH 7.4).

### Pharmacological Agents and Reagents

Carbachol; DA hydrochloride; the non-competitive NMDAR antagonist, (5*S*,10*R*)-(+)-5-methyl-10,11-dihydro-5*H*-dibenzo[*a*,*d*]cyclohepten-5,10-imine or dizocilpine hydrogen maleate (MK-801); and the PI3K inhibitor, 11-(acetyloxy)-1*S*,6*bR*,7,8,9*aS*,10,11*R*,11*bR*-octahydro-1-(methoxymethyl)-9*a*,11*b*-dimethyl-3*H*-furo[4,3,2-*de*]indeno[4,5-*h*]-2-benzopyran-3,6,9-trione (wortmannin), were purchased from Tocris Cookson Ltd. (Bristol, United Kingdom). All other drugs and ACSF salts were purchased from Sigma-Aldrich (Poole, United Kingdom). Stock solutions, at thousand times the final concentration, were made in water or DMSO and stored in individual aliquots at −20°C. The final solutions were freshly prepared on the day of the experiment.

### Electrophysiological Recording, Data Acquisition, and Statistical Analysis

The hippocampal slices were maintained at a temperature of 32°C at the interface between the ACSF and warm humidified carbogen and allowed to equilibrate in this medium for 1 h prior to recording. Extracellular field potentials were recorded from the stratum pyramidale of Cornu ammonis 3c (CA3c) of the hippocampus, using glass microelectrodes containing ACSF (resistance, 2–5 MΩ). Field potentials were amplified using NeuroLog NL106 AC/DC amplifiers (Digitimer Ltd., Welwyn Garden City, United Kingdom) and band-pass filtered between 0.5 and 500 Hz using NeuroLog NL125 filters (Digitimer Ltd., Welwyn Garden City, United Kingdom). Electromagnetic interference from the main supply was eliminated from the recordings with the use of HumBug 50-Hz noise eliminators (Digitimer Ltd., Welwyn Garden City, United Kingdom). The recordings were digitized at a sample rate of 2 kHz using a CED 1401-plus ADC board (Cambridge Electronic Design, Cambridge, United Kingdom).

Data were analyzed offline using the Spike2 software (Cambridge Electronic Design). Power spectra were generated to provide a quantitative measure of the frequency components. Power spectra were constructed for 60-s epochs using a fast Fourier transform algorithm.

It has been widely accepted that *in vitro* γ oscillations ranged from 20 to 80 Hz, because the recorded γ oscillations in brain slices are temperature dependent and the slice recordings performed mostly at 32°C rather than 37°C. There is a linear relationship between peak frequency of network oscillations and temperature, in which an increase of 1°C in temperature of brain slices corresponds to an increase of 2.3 ± 0.4 Hz in the oscillation frequency (Dickinson et al., 2003; Lu et al., 2012).

The area under the curve between 20 and 60 Hz was used to quantify the γ power. Autocorrelograms were calculated in Spike2 using a 500-ms lag from the same local field potential trace used for γ power calculation. The decay time constant (tau) of the autocorrelation peaks is a measure of the regularity of the oscillation and generated by fitting the autocorrelation peaks with an exponential function: *Y* = exp(−*a* ^∗^ *X*).

### Statistical Analysis

All statistical analyses were performed using IBM SPSS Statistics 22 software (IBM, Armonk, NY, United States). The Shapiro–Wilk test was used in testing the normality of the data. Parametric data were expressed as mean ± standard error of the mean. The paired and unpaired Student’s *t*-tests were used to compare two groups of parametric data. One-way analysis of variance (ANOVA) and repeated-measures (RM) ANOVA were used to compare three or more group means. Non-parametric data were expressed as median ± interquartile range. The Wilcoxon rank-sum and signed-rank tests were used to compare the two groups of non-parametric data. One-way and RM ANOVAs on ranks were used for three or more group comparisons. The parametric two-way ANOVA was used to analyze experimental data derived from two-factor designs with or without RM. The two-way ANOVA on ranks was used to analyze non-parametric data. A *P*-value < 0.05 was considered statistically significant.

## Results

### Gamma Oscillations Were Intact in Heterozygous Reeler Mice Compared With Wild-Type Mice

**FIGURE 1:**
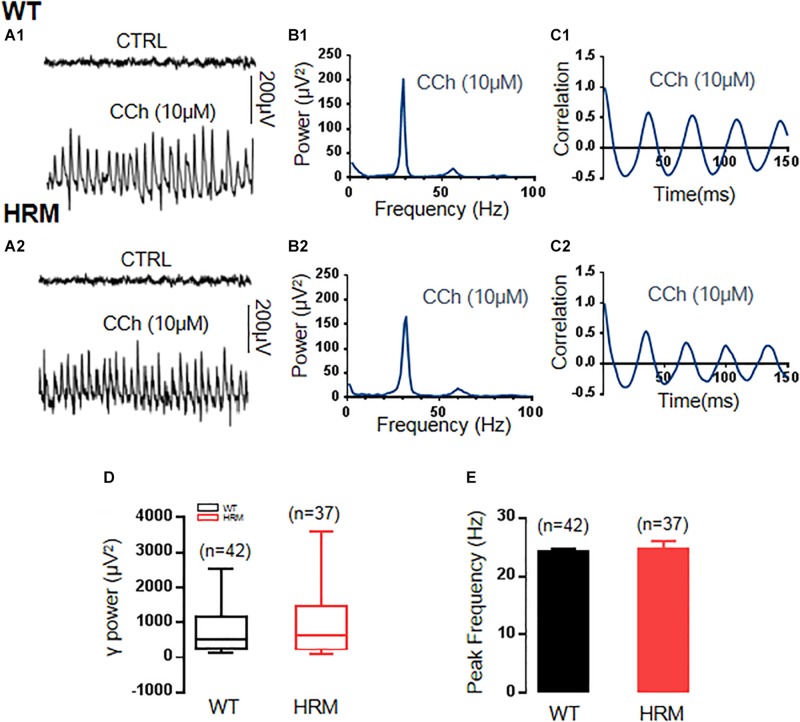
γ power in hippocampal CA3 region in wild-type mice (WTM) and heterozygous reeler mice (HRM). **(A1,A2)** The original curve of 1-s field potential induced by carbachol (CCh) recorded in the hippocampal CA3 region in WTM **(A1)** and HRM **(A2)** hippocampal slices. **(B1,B2)** The power spectrum of field potential induced by CCh from WTM **(B1)** and HRM **(B2)** hippocampal slices. **(C1,C2)** Autocorrelograms of the recordings in **A1** and **A2** show the oscillation regularity of CCh-induced oscillations from WTM **(C1)** and HRM **(C2)**. **(D)** The bar graph shows the values of γ power of CCh-induced oscillations in WTM and HRM. **(E)** The peak frequency of CCh-induced oscillations in WTM and HRM.

To induce stable γ oscillations in the CA3 area of mouse hippocampal slices, the cholinergic agonist carbachol at 10 μM, half of the concentration used in γ induction in rat hippocampal slices (Fisahn et al., 1998), has been applied in bath perfusion. The γ oscillations were induced after 5–10 min of an application of carbachol in hippocampal slices, gradually increased, and reached the steady state in approximately 1–2 h. Sample traces of field potentials of baseline (no carbachol) and carbachol-induced γ oscillations are presented in Figures 1A1,A2. In the comparison between WTM and HRM, there was no significant difference in γ power [WTM, 526.97 (247.21, 1,140.19) μV^2^, *n* = 45 slices from 22 mice, vs. HRM, 646.30 (239.25, 1,374.71) μV^2^, *n* = 37 slices from 19 mice, Mann–Whitney *U-*statistic = 744, *T* = 1,513.00, *P* = 0.749, Figures 1B1,B2,D] and peak frequency of oscillations [WTM, 24.3 ± 0.55 Hz; HRM, 24.9 ± 1.4 Hz; *t*(28) = 0.373; *P* = 0.712; Figure 1E]. Carbachol-induced γ oscillations were regular in both WTM and HRM, reflected by the similar decay time constants generated by fitting autocorrelation curves with an exponential function [116.6 ± 13.2 ms for WTM vs. 129.7 ± 10.8 ms for HRM, *t*(12) = 0.726, *P* = 0.482, Figures 1C1,C2].

### Dopamine Increased Gamma Power in Wild-Type Mice but Not in Heterozygous Reeler Mice

**FIGURE 2:**
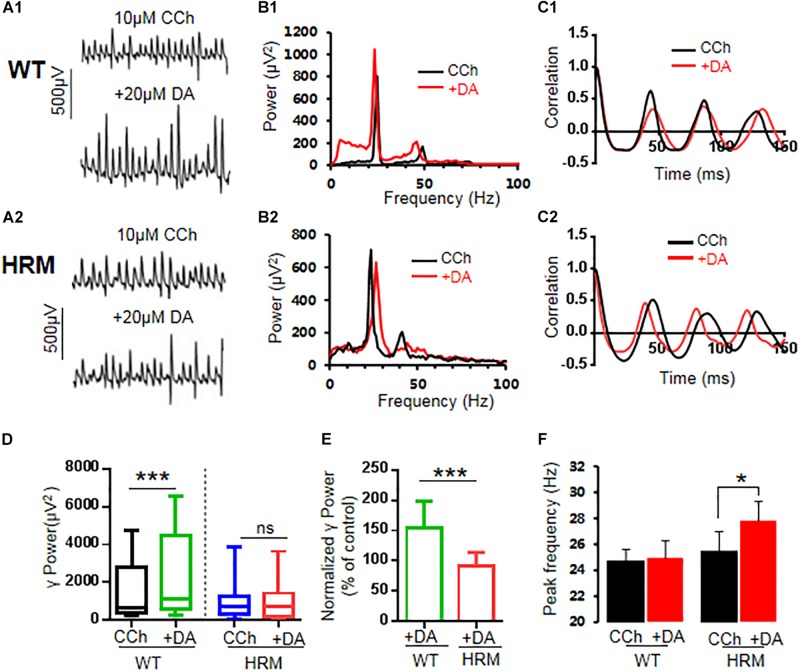
Effect of dopamine (DA) on γ oscillations in slices of hippocampal CA3 from wild-type mice (WTM) and heterozygous reeler mice (HRM). The original curve of 1-s field potential before and after application of DA (20 μM) recorded in slices of hippocampal CA3 region from WTM **(A1)** and HRM **(A2)**. **(B1,B2)** The power spectrum of field potential before and after application of DA (20 μM) from WTM **(B1)** and HRM **(B2)** hippocampal slices. **(C1,C2)** Autocorrelograms of the recording in **A1** and **A2** show the effects of DA on oscillation regularity from WTM **(C1)** and HRM **(C2)**. **(D)** The bar graph shows the effects of carbachol (CCh) and CCh + DA on γ power in WTM and HRM (***P < 0.001). **(E)** The bar graph shows the effects of DA (CCh + DA) on normalized γ power (percentage change over control) in WTM and HRM (***P < 0.001). **(F)** The bar graph shows peak frequency of the oscillatory activity in WTM and HRM (*P < 0.05).

Defective reelin signaling influences the mesolimbic dopaminergic pathways (Pujadas et al., 2014). Thus, we tested whether DA modulation of γ oscillations was altered in HRM. After stable γ oscillations were induced by carbachol in hippocampal CA3 for at least 30 min, 20 μM of DA was applied. In WTM, DA increased the γ power by 53.8 ± 11.5% of the control [CCh + DA, 1,095.24 (586.52, 3,932.55) vs. CCh, 650.83 (392.70, 2,750.91) μV^2^, *Z*-statistic = 3.233, *n* = 14 slices from six mice, *P* < 0.001, Wilcoxon signed-rank test, Figures 2A1,B1,C1,D]. However, DA had no effect on γ power in HRM [CCh + DA, 703.43 (214.68, 1,369.28) vs. CCh, 727.58 (387.55, 1,223.66) μV^2^, *Z*-statistic = −1.363, *n* = 13 slices from five mice, *P* = 0.191, Wilcoxon signed-rank test, Figures 2A2,B2,C2,D]. There was a significant difference in DA response between WTM and HRM [*t*(25) = 4.626, *P* = 0.0001, *t*-test, Figure 2E]. A two-way non-parametric ANOVA for γ powers revealed a significant main effect of genotype (*F*_(__1_,_25__)_ = 25.559, *P* < 0.0001) and a significant main effect of 20 μM of DA (*F*_(__1_,_25__)_ = 5.279, *P* = 0.026). Moreover, there was a significant interaction effect between genotype and 20 μM of DA (*F*_(__1_,_25__)_ = 25.559, *P* < 0.0001). These results indicate that DA increased γ power in WTM but not in HRM.

Dopamine had no effect on peak frequency in WTM [CCh + DA, 24.9 ± 1.3 Hz vs. CCh, 24.7 ± 1.9 Hz; *t*(7) = −0.287, *P* = 0.783, paired *t*-test] but slightly increased peak frequency in HRM [CCh + DA, 27.8 ± 1.5 Hz vs. CCh, 25.5 ± 1.4 Hz, *t*(7) = −2.600, *P* = 0.035, paired *t*-test, Figure 2F]. The regularity of γ oscillations was not altered by DA in both WTM and HRM, as the time constants are similar before and after the application of DA in either WTM [CCh, 116.6 ± 13.2 ms vs. CCh + DA, 129.1 ± 26.7 ms, *t*(7) = 0.544, *P* = 0.603, paired *t*-test] or HRM [CCh, 129.7 ± 15.6 ms vs. CCh + DA, 103.0 ± 8.1 ms, *t*(5) = −1.768, *P* = 0.137, paired *t*-test].

Previous studies indicate that a high DA concentration (200 μM) actually inhibited CCh-induced γ oscillations in rat hippocampus (Weiss et al., 2003). Thus, we tested the effects of DA on γ oscillations at such a high concentration in both WTM and HRM. Our results show that DA significantly enhanced γ power [1,431.84 ± 408.54 μV^2^ vs. CCh, 911.55 ± 27.07 μV^2^, *t*(5) = −3.235, *P* = 0.023] without affecting peak frequency [24.47 ± 1.22 Hz vs. CCh, 25.3 ± 0.78 Hz, *t*(5) = 1.398, *P* = 0.221] in WTM and had no significant effect on either γ power [910.69 (574.66, 1,067.17) μV^2^ vs. CCh, 892.46 (514.53, 1,005.38) μV^2^, *Z*-statistic = 1.859, *P* = 0.078, Wilcoxon signed-rank test] or peak frequency [24.41 ± 1.26 Hz vs. CCh, 24.07 ± 137 Hz, *t*(6) = −0.67, *P* = 0.518] in HRM. A two-way non-parametric ANOVA for γ powers revealed a significant main effect of genotype (*F*_(__1_,_38__)_ = 33.749, *P* < 0.0001) and no significant main effect of DA concentrations (*F*_(__1_,_38__)_ = 1.925, *P* = 0.174). There was no significant interaction effect between genotype and DA concentrations (*F*_(__1_,_38__)_ = 0.896, *P* = 0.350).

### MK-801 Increased Gamma Power and Occluded the Effect of Dopamine on Gamma Power in Wild-Type Mice

**FIGURE 3:**
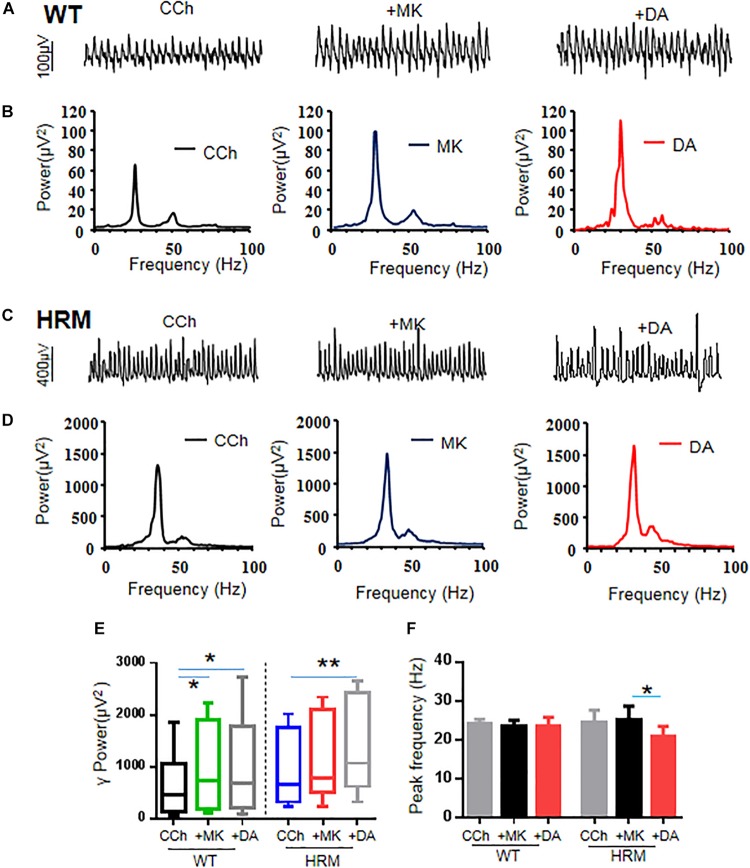
Effect of MK-801 on γ oscillations and dopamine (DA)-mediated γ oscillations in hippocampal CA3 of wild-type mice (WTM) and heterozygous reeler mice (HRM). **(A,C)** The original curves of 1-s field potentials after applications of carbachol (CCh), MK-801, and DA recorded in hippocampal CA3 region in WTM **(A)** and HRM **(C)**. **(B,D)** The power spectra of field potentials after application of CCh, MK-801, and MK-801 + DA in WTM **(B)** and HRM **(D)**. **(E)** The bar graph shows the effects of CCh, MK-801, and Mk-801 + DA on γ power in WTM and HRM (*P < 0.05, **P < 0.01). **(F)** The bar graph shows the effects of CCh, MK-801, and Mk-801 + DA on the peak frequency of oscillatory activity recorded in CA3 region of hippocampal slices in WTM and HRM (*P < 0.05).

Because NMDAR antagonists can restore dendritic spine density and synaptic plasticity in the early stages in HRM (Niu et al., 2004), we examined the effect of NMDAR antagonist on γ oscillations in WTM. Perfusion of hippocampal slices of WTM with MK-801 (20 μM) significantly increased γ power by 54.5 ± 10.9% of control [CCh + MK-801, 737.23 (178.1, 1,756.03) μV^2^ vs. CCh, 459.55 (143.27, 971.16) μV^2^, *q* = 4.648, *P* < 0.05, RM ANOVA on ranks, followed by Tukey’s test, Figures 3A,B,E]. A further application of DA (20 μM) did not significantly change the γ power [CCh + MK-801 + DA, 685.56 (218.2, 1,666.23) μV^2^ vs. CCh + MK-801, 737.23 (178.1, 1,756.03) μV^2^, *q* = 1.549, *P* > 0.05, RM ANOVA on ranks, followed by Tukey’s test, Figures 3A,B,E], suggesting that MK-801 occluded the effect of DA on γ power in WTM. The effect of MK-801 + DA on γ power was not different from that of DA alone [*t*(27) = 0.758, *P* = 0.455, *t*-test], and the net increase of γ power caused by DA after deducting the effect of MK-801 was significantly smaller than that of DA alone in WTM [8.9 ± 5.1% vs. DA, 53.8 ± 11.9%, *t*(27) = −3.535, *P* = 0.001, *t*-test]. Neither MK-801 nor MK-801 + DA had any effect on the peak frequency in WTM (Figure 3F).

### MK-801 Caused a Small Increase in Gamma Power and Restored Dopamine-Mediated Enhancement of Gamma in Heterozygous Reeler Mice

Perfusion of hippocampal slices of HRM with MK-801 (20 μM) caused a 24.6 ± 11.3% change in the γ power without statistical significance [CCh + MK-801, 780.23 (607.33, 2,037.99) μV^2^ vs. CCh, 646.30 (327.35, 1,650.56) μV^2^, *q* = 3.000, *P* > 0.05, RM ANOVA on ranks, followed by Tukey’s test, Figures 3C–E]. A two-way non-parametric ANOVA for γ powers revealed a significant interaction effect between genotype and MK-801 (*F*_(__1_,_22__)_ = 4.618, *P* = 0.037). A further application of DA (20 μM) caused an additional 21.3 ± 3.8% increase (over MK-801) or a total increase of 50.4 ± 13.3% (over CCh) on γ power [1,065.32 (726.59, 2,317.82) μV^2^ vs. CCh, 646.30 (327.35, 1,650.56) μV^2^, *q* = 6.000, *P* < 0.05, RM ANOVA on ranks, followed by Tukey’s test, Figures 3C–E]. Compared with DA alone in HRM, MK-801 + DA caused a significant increase on γ power in HRM [50.4 ± 13.3% vs. DA, −8.6 ± 6.3%, *t*(21) = 2.930, *P* = 0.008, *t*-test], and such an increase was comparable with that of DA in WTM (DA, 53.8 ± 11.9%). The net increase of γ power caused by DA after deducting the effect of MK-801 was also significantly larger than that of DA alone in HRM [21.3 ± 3.8% vs. DA, −8.6 ± 6.3%, *t*(20) = 3.64, *P* = 0.002, *t*-test]. These results suggest that MK-801 restored partial sensitivity of γ power to DA in HRM despite the fact that the effect of MK-801 on γ power in HRM is significantly less than that of MK-801 in WTM (HRM, 24.6 ± 11.3% vs. WTM, 54.5 ± 10.9%, Mann–Whitney *U*-test = 101.000, *T* = 79, *P* = 0.049, Mann–Whitney rank-sum test).

Interestingly, neither MK-801 alone nor MK-801 + DA had any effect on peak frequency of oscillations in WTM (CCh + MK-801, 23.4 ± 1.6 Hz vs. CCh, 24.3 ± 1.0 Hz or vs. CCh + MK-801 + DA, 23.6 ± 2.2 Hz, *F*_(__2_,_5__)_ = 0.333, *P* = 0.726, RM ANOVA, Figure 3F). In HRM, MK-801 alone had no effect on peak frequency of oscillations (CCh + MK-801, 25.2 ± 3.5 Hz vs. CCh, 24.6 ± 3.2 Hz) and blocked the increasing effect of DA on peak frequency and actually reduced peak frequency to 20.9 ± 2.6 Hz (CCh + MK-801 + DA) from 25.2 ± 3.5 Hz (CCh + MK-801) (RM ANOVA, *F*_(__2_,_4__)_ = 5.481, *P* = 0.032, followed by the Holm–Sidak method). These results suggest that NMDAR antagonist reversed the effect of DA on oscillatory peak frequency in HRM.

### Wortmannin Increased Gamma Power and Largely Blocked Dopamine-Mediated Increase in Gamma Power in Wild-Type Mice

**FIGURE 4:**
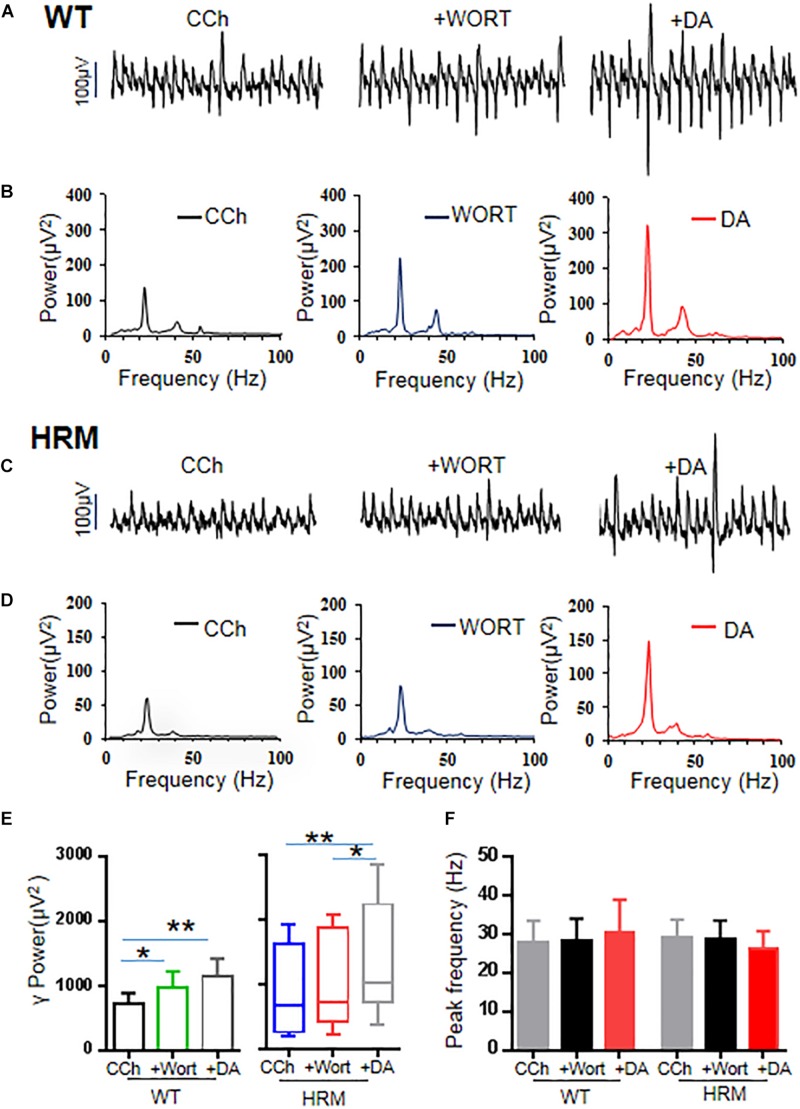
Effect of wortmannin on γ oscillations and dopamine (DA)-mediated γ oscillations in hippocampal CA3 of wild-type mice (WTM) and heterozygous reeler mice (HRM). **(A,C)** The original curves of 1-s field potentials after application of carbachol (CCh), wortmannin, and wortmannin + DA recorded in hippocampal CA3 from WTM **(A)** and HRM **(C)**. **(B,D)** The power spectra of field potentials after application of CCh, wortmannin, and wortmannin + DA recorded in hippocampal CA3 from WTM **(B)** and HRM **(D)** hippocampal slices. **(E)** The bar graph shows the effects of wortmannin and wortmannin + DA on γ power in WTM and HRM (*P < 0.05, **P < 0.01). **(F)** The bar graph shows the effects wortmannin and wortmannin + DA on peak frequency of oscillatory activity recorded in hippocampal CA3 from WTM and HRM.

Previous studies indicate that reelin acts on its receptor and activates the PI3K–Akt–mammalian target of rapamycin (mTOR) pathway (Hwa and Gabriella, 2016). Therefore, we examined the effect of wortmannin, a potent and selective inhibitor of PI3K, at a physiological dose (Wang et al., 2017) on γ oscillations of rat hippocampal slices from WTM and HRM. When wortmannin was applied to hippocampal slices, γ power was significantly increased by 39 ± 12% in WTM (CCh + Wort, 973.39 ± 252.78 μV^2^ vs. CCh, 715.89 ± 175.34 μV^2^, *F*_(__2_,_9__)_ = 9.908, *P* = 0.001, RM ANOVA, Figures 4A,B,E), and a further application of DA (20 μM) caused an additional 23 ± 7% increase in γ power, but such an increase did not reach statistical significance compared with that in wortmannin (1,150.03 ± 273.81 μV^2^ vs. CCh + Wort, *T* = 1.801, *P* = 0.09, RM ANOVA, followed by the Holm–Sidak method, Figures 4A,B,E). These results indicate that wortmannin largely blocked DA-mediated enhancement of γ power in WTM. Neither wortmannin nor wortmannin + DA had any effect on peak frequency of γ oscillations in WTM (Figure 4F).

### Wortmannin Restored Dopamine Response of Gamma Power in Heterozygous Reeler Mice

When applied to hippocampal slices from HRM, wortmannin (200 nM) increased γ power by 20 ± 7.5% without statistical significance [716.87 (426.0, 1,829.91) μV^2^ vs. CCh, 673.45 (269.47, 1,605.95) μV^2^, *q* = 2.53, *P* > 0.05, Friedman RM ANOVA on ranks, followed by *post hoc* Tukey’s test, Figures 4C,D,E]. A further application of DA (20 μM) caused an additional 46 ± 13% increase in γ power [1,022.7 (784.16, 2,150.97) μV^2^ vs. wortmannin, *q* = 3.479, *P* < 0.05; vs. CCh, *q* = 6.008, *P* < 0.05, Friedman RM ANOVA on ranks (Friedman statistic = 18.20, *P* < 0.001), followed by *post hoc* Tukey’s test, Figures 4C,D,E]. A two-way non-parametric ANOVA for γ powers revealed no significant interaction effect between genotype and wortmannin (*F*_(__1_,_18__)_ = 0.572, *P* = 0.454) and no significant interaction effect between genotype and Wort + DA (*F*_(__1_,_18__)_ = 0.396, *P* = 0.533). With the effect of DA alone on γ power (−8.6 ± 6.8%) in HRM, such an increase of 46 ± 13% in γ power is of statistical significance [*t*(21) = −4.109, *P* = 0.001, *t*-test]. DA mediated an increase in γ power in the presence of wortmannin in HRM at a level that is comparable with that of DA effect on γ power in WTM, which suggests that wortmannin restored the response of hippocampal γ oscillations to DA in HRM. Neither wortmannin nor wortmannin + DA had any effect on peak frequency of γ oscillations in HRM (Figure 4F).

## Discussion

Our main findings are as follows: (1) DA enhanced γ power in WTM but not in HRM. (2) MK-801 induced a larger increase in γ power, occluded the effect of DA in WTM, induced a small increase in γ power, and partially restored the effect of DA in HRM. (3) Wortmannin induced a larger increase in γ power, blocked the effect of DA in WTM, and caused no significant increase in γ power but largely restored the effect of DA in HRM.

### Altered Dopamine Modulation of Hippocampal Gamma Oscillation in Heterozygous Reeler Mice

Dopamine at a concentration of 20 or 200 μM increased γ power in hippocampal slices in WTM, which differs from the observation that DA at a concentration of 200 μM reduced γ oscillations induced by carbachol in area CA3 of rat hippocampus (Weiss et al., 2003), suggesting that species difference may exist in DA modulation of γ oscillations.

In HRM, we demonstrated that *in vitro* hippocampal γ oscillation was intact in HRM but that DA modulation of γ oscillations was impaired. Loss of sensitivity to DA for hippocampal γ power in HRM may be related to altered densities of dopaminergic fibers in different brain areas: increased in the hippocampus but reduced in the ventral tegmental area and nucleus accumbens in HRM (Nullmeier et al., 2014). The expression profile of DA receptors in the hippocampus of HRM is relatively sparse but decreased D1 and D2 receptors in the striatum (Matsuzaki et al., 2007) or altered expression pattern of D2R in different brain areas: An increased expression in the striatum but decreased expression in the frontal cortex (Varela et al., 2015) was reported.

### Blocking *N*-Methyl-D-Aspartate Receptor Partially Restores Dopamine Sensitivity in Heterozygous Reeler Mice

Clinical symptoms of schizophrenia are associated with altered cortical neuronal oscillations in γ rhythms. NMDAR antagonists induce psychotic symptoms in humans and a schizophrenia-like phenotype in animals (Spencer, 2011; Jadi et al., 2016). In this study, NMDAR antagonist increased γ power in the hippocampal slices of WTM, which is in agreement with a previous study that a single application of the NMDAR antagonist MK-801 in rats increased the power and reduced the peak frequency of γ oscillations (Lemercier et al., 2017).

Compared with WTM, the same dose of MK-801 caused a relative small increase in γ power in HRM, which may be related to the possible alteration in the composition of NMDAR subunits in the hippocampus. It was reported that blocking reelin secretion increases the NR2B subunit in cultured hippocampal neurons (Campo et al., 2009). HRM also showed increased NR1 but reduced NR2C in the frontal cortex (van den Buuse et al., 2012). Additionally, during neural maturation, a marked decrease in NR1/NR2B receptor participation to NMDAR-mediated synaptic currents concomitant with the accumulation of reelin at active synapse was observed in cultured hippocampal neurons, suggesting that reelin regulates NMDAR surface trafficking and synaptic subunit composition (Sinagra et al., 2005; Groc et al., 2007). Reelin also regulates NMDAR function via increased tyrosine phosphorylation of NR2A and NR2B receptors and increases NMDAR-mediated synaptic plasticity in the adult hippocampus (Qiu et al., 2006). These studies indicate that sufficient reelin is required to control the subunit composition and function of NMDAR in hippocampal neurons and that reelin deficiency causes altered composition and reduced function of NMDAR, which will likely contribute to the altered response of γ oscillation to MK-801.

In WTM, MK-801 occluded DA-mediated increase in γ power, indicating that DA enhancement of γ oscillation is through NMDAR activation. This is in agreement with previous reports that DA-mediated (Andersson et al., 2012), nicotine-mediated (Wang et al., 2017), and methamphetamine-mediated (Li et al., 2019) increase in γ oscillation in rat hippocampus are all involved in NMDAR activation.

In HRM, MK-801 partially restored DA-mediated response of γ oscillation. The explanation for this result could be that blunted DA modulation of γ oscillations by overactivation of NMDAR in HRM may be attenuated by MK-801, as observed in the case that intensive NMDAR activation mediated nicotine (100 μM) inhibition of γ oscillations (Wang et al., 2017). However, reduced NMDAR-dependent synaptic long-term potentiation in HRM (Iafrati et al., 2014) suggests that NMDAR activity may be at a relative low level in HRM. Although detailed mechanisms for the partial restoration of DA enhancement of γ power remain to be further studied, our results are supported by the observation that NMDAR antagonists, ketamine or Ro25-6981 (selective inhibitor of GluN2B), restored synaptic and memory function in HRM (Iafrati et al., 2014). The similar roles between Ro25-6981 and ketamine in HRM imply that correcting NMDAR composition from an immature form (GluN2B) to mature form (GluN2A) is important in recovering normal synaptic transmission in HRM. One study showed that MK-801 altered subunits of NMDAR in the young adult rat prefrontal cortex (Xi et al., 2009), although it is not known whether MK-801 affects the composition of NMDARs in the hippocampus in HRM. Interestingly, MK-801 and ketamine not only alter NMDAR composition but also have a partial agonist effect on D2 receptor (Kapur and Seeman, 2002), which may be critical in DA modulation of γ power, especially in HRM.

Dopamine alone increased the peak frequency of oscillatory activity in HRM. This effect was reversed, whereas DA effect on γ power was partially restored in the presence of MK-801, which suggests that NMDAR activation is required for DA-mediated oscillatory frequency.

### Blocking Phosphatidylinositol 3-Kinase Largely Restores the Dopamine Sensitivity in Heterozygous Reeler Mice

Similar to the effects of MK-801 on γ oscillations, wortmannin, a PI3K inhibitor, caused a substantial increase in γ power in WTM and a small, insignificant increase in HRM, indicating that the endogenous PI3K activity is different between WTM and HRM and that sensitivity of γ oscillations to PI3K activity is reduced in HRM.

In WTM, wortmannin was able to occlude DA enhancement of γ power, which indicates that PI3K is also involved in DA modulation of γ oscillation. In HRM, DA-mediated response was largely increased in the presence of wortmannin. Our result is in agreement with the report that blocking NMDAR and its downstream signaling molecule, the mTOR, rescued the deficit of function and behavior in HRM (Iafrati et al., 2014). Studies also demonstrated that reelin, acting through the PI3K, positively modulates the activity of mTOR kinase, which is required in the stimulation of dendrite outgrowth, and activates downstream proteins, such as the p70S6K, which are known to participate in the control of protein translation (Jossin and Goffinet, 2007; Ventruti et al., 2011). Because PI3K is an upstream signaling molecule of mTOR (Lussier et al., 2016) and a downstream molecule of NMDAR (Perkinton et al., 2002; Man et al., 2003; Crossthwaite et al., 2004), it is reasonable to assume that the restoration of DA enhancement of hippocampal γ oscillations in HRM in the presence of wortmannin is likely through inhibition of the NMDAR–PI3K signaling pathway.

As reelin activates PI3K (Beffert et al., 2002) and enhances synaptic transmission via PI3K-dependent synaptic insertion of AMPARs in adult hippocampus (Qiu et al., 2006), HRM with remarkable reelin deficiency may have a low level PI3K activity, which may explain the blunted response of γ oscillations to PI3K inhibitor. However, it is unclear how DA modulation of γ oscillations is largely recovered in the presence of a PI3K inhibitor in HRM. Although the mechanism of this observation remains to be further determined, our results with respect to the large restoration of DA sensitivity in the presence of PI3K inhibitor nevertheless indicate a possibility on how to correct abnormality in DA function in HRM.

The results of this study demonstrated that the altered DA modulation of γ oscillations in HRM is associated with dysregulated NMDAR–PI3K signaling, establishing a link between DA- and NMDAR-mediated signaling, network oscillations, and reelin, which might be relevant to the field of schizophrenia research.

## Data Availability Statement

The raw data supporting the conclusions of this manuscript will be made available by the authors, without undue reservation, to any qualified researcher.

## Ethics Statement

The animal study was reviewed and approved by the Animal Experimentation Ethics Committee of Xinxiang Medical University (protocol number: 11401300017419). All experiments were performed in accordance with the guidelines of the Animal Care and Use Committee of Xinxiang Medical University.

## Author Contributions

LW, DZ, MW, and YW performed the research. CL and JL designed the research. CL, LW, DZ, and MV analyzed the data. LW, DZ, and CL wrote the manuscript. CL, LW, and MV revised the manuscript. All authors approved the final manuscript for publication.

## Conflict of Interest

The authors declare that the research was conducted in the absence of any commercial or financial relationships that could be construed as a potential conflict of interest.
